# Feasibility of minimally invasive stimulation of the phrenic nerves for supporting ventilation in fully anesthetized swine

**DOI:** 10.1186/cc12069

**Published:** 2013-03-19

**Authors:** J Bijwadia, M Karamanoglu

**Affiliations:** 1 Regions Hospital, St Paul, MN, USA; 2 Respithera, Bloomington, MN, USA

## Introduction

Long-term use of mechanical ventilators may lead to ventilator-induced diaphragmatic dysfunction (VIDD) and increase the duration of weaning from MV [1]. It was hypothesized that stimulating the diaphragm during MV may prevent VIDD and may lead to early weaning [2]. In this study, the feasibility of generating coordinated contraction of both diaphragms was investigated using a novel transvenous diaphragmatic pacing system.

## Methods

Two juvenile pigs were anesthetized with propofol (150 to 250 μg/kg/minute) and ventilated (VENT) with an assist control mode MV (Nellcor Puritan Bennett 840). Using fluoroscopy, a novel multipolar neurostimulation catheter (Inspirx RL PICC53; Respithera, Bloomington, MN, USA) was threaded into the left internal jugular vein and advanced to the junction of right atrium and the superior vena cava using a modified Seldinger technique. The successful capture of the right and left phrenic nerves was confirmed by fluoroscopic visualization. Peak airway pressures (PAWP) and blood gases were determined after 10 minutes MV (MV), MV and stimulation applied together (MV+STIM) and stimulation only (STIM).

## Results

No animal-ventilator dyssynchrony during stimulation (MV+STIM) was noted while peak airway pressures were reduced. During STIM there was no discernible paradoxical movement of the diaphragm. In addition, PCO_2 _and PO_2 _confirmed that adequate ventilation and oxygenation can be provided by the system, while PAWP could be reduced (Table 1).

**Table 1:** Ventilation parameters

	MV	MV+STIM	STIM
PAWP (cmH_2_O)	20	16	9
PO_2 _(mmHg)	89	101	95
PCO_2 _(mmHg)	32	26	26

## Conclusion

It was possible to capture and stimulate both phrenic nerves using a minimally invasive approach to support respiration and sustain blood gases at physiological levels. This development could help wean MV-dependent ICU patients earlier. Further long-term studies are needed to assess the full potential of this novel system.
